# Chondrotoxicity of Intra-Articular Injection Treatment: A Scoping Review

**DOI:** 10.3390/ijms25137010

**Published:** 2024-06-26

**Authors:** Carmelo Pirri, Andrea Sorbino, Nicola Manocchio, Nina Pirri, Antonio Devito, Calogero Foti, Alberto Migliore

**Affiliations:** 1 Department of Neurosciences, Institute of Human Anatomy, University of Padova, 35121 Padova, Italy; carmelo.pirri@unipd.it; 2 Physical and Rehabilitation Medicine, Department of Clinical Sciences and Translational Medicine, Tor Vergata University, 00133 Rome, Italy; a.sorbino@quaderni.biz (A.S.); nicola.manocchio@uniroma2.it (N.M.); foti@med.uniroma2.it (C.F.); 3 Department of Medicine—DIMED, School of Radiology, Radiology Institute, University of Padova, 35121 Padova, Italy; nina_92_@hotmail.it; 4 Internal Medicine, S. Pietro Fatebenefratelli Hospital, 00189 Rome, Italy; antonio.devito84@icloud.com; 5 Rheumatology, S. Pietro Fatebenefratelli Hospital, 00189 Rome, Italy

**Keywords:** chondrotoxicity, intra-articular injection, corticosteroids, local anaesthetic, nonsteroidal anti-inflammatory drugs, hyaluronic acids, platelet-rich plasma (PRP)

## Abstract

The purpose of this scoping review was to identify possible chondrotoxic effects caused by drugs usually used for intra-articular injections. PubMed, Scopus, Web of Science and Cochrane were searched. Inclusion criteria required randomized controlled trials written in English that evaluate the toxic effect that damages the cartilage. The literature search resulted in 185 unique articles. 133 full-text articles were screened for inclusion, of which 65 were included. Corticosteroids, with the exception of triamcinolone, along with local anaesthetics, potentially excluding ropivacaine and liposomal bupivacaine, and nonsteroidal anti-inflammatory drugs, exhibited insufficient safety profiles to warrant casual use in clinical settings. Hyaluronic acid, on the other hand, appears to demonstrate safety while also mitigating risks associated with concurrent compounds, thereby facilitating therapeutic combinations. Additionally, there remains a paucity of data regarding platelet-rich plasma, necessitating further evaluation of its potential efficacy and safety. Overall, it seems that results are significantly influenced by the dosage and frequency of injections administered, observed in both human and animal studies.

## 1. Introduction

Several studies have highlighted the extensive use of intra-articular (IA) injections in treating various forms of arthritis. This includes a specific focus on osteoarthritis (OA), as well as other inflammatory and rheumatologic conditions, such as rheumatoid arthritis and psoriatic arthritis [1]. For example, OA is a degenerative disease of the cartilage, associated with important changes in cartilage metabolism. If an imbalance between degradation and synthesis by chondrocytes occurs, OA may appear. In fact, inactivity has been shown to lead to the degradation of cartilage and joint motion has a high significance in maintaining cartilage physiological properties [2]. IA injection treatments are not only used to manage diseases in the acute phase but even as chronic therapy, leading to the chance of reducing long-term consequences [3]. The most used medications for IA injections are corticosteroids, local anaesthetics, hyaluronic acids (HA), platelet-rich plasma (PRP), nonsteroidal anti-inflammatory drugs (NSAIDs), collagen medical devices and bisphosphonates [4,5]. Like all substances used as drugs, these compounds can also have side effects. This is particularly so for the joint environment, as it is particularly delicate. In this regard, articular cartilage is a balanced tissue of 2–4 mm-thick hyaline cartilage with no nerves and blood or lymphatics vessels, comprising a complex extracellular matrix (ECM) and specialized cellular elements. This sophisticated composition confers upon it the capacity to endure substantial compressive forces while enabling smooth, frictionless articulation of the joints.

In this context, ECM is the main structure of articular cartilage. Water, collagen, proteoglycans, other non-collagenous proteins and glycoproteins are the main ECM constituents [6,7]. The amount of water present within ECM is critical to maintain its unique mechanical properties and is determined by the aforementioned structural components [8]. The ideal quota of tissue fluid should be between 65% and 80% of the total weight [9]. Considering the dry weight of cartilage, collagen alone accounts for about 60% of the total, making it the most represented molecule. Fibers and fibrils of type II collagen (90%) are stabilized by other less frequently appearing collagen types and are associated with proteoglycan [10]. Another 10–15% of the dry weight is constituted by proteoglycans, heavily glycosylated protein monomers. The largest and most copious is called aggrecan; it has a peculiar ability to interact with HA to form large aggregates and provide cartilage with osmotic properties, needed to resist compressive loads [11]. The remaining dry weight is made of other proteins and glycoproteins (e.g., fibronectin) which may play a role in the organization and maintenance of the structure of the ECM [12].

The resident population of cells is the chondrocytes. Chondrocytes, highly specialized and metabolically active cells, have a distinctive function in the formation, upkeep, and restoration of the ECM. They derive from mesenchymal stem cells and make up approximately 2% of the overall volume of articular cartilage [13]. Chondrocytes create specialized microenvironments and regulate the turnover of the ECM in their vicinity. Chondrocytes are trapped within their microenvironment’s matrix, hindering migration and direct cell-to-cell contacts. However, they can respond to various stimuli (e.g., growth factors and mechanical forces). Chondrocytes have limited replication capacity, meaning that, consequently, cartilage has low healing potential after damage of any kind, and cell survival is dependent on optimal chemical and mechanical conditions [7].

The role of chondrocytes in ECM and articular cartilage is essential. They can synthesize ECM components and several enzymes responsible for its remodelling. As vascularization is lacking in articular cartilage, and thus oxygen and nutrients are scarce, chondrocytes have a primarily anaerobic metabolism. Their metabolic activity can be altered by a variety of factors, such as cytokines, growth factors, regulatory peptides, biomechanical forces, joint motion and load [14].

Considering all of this, it is clear that articular cartilage is a particularly delicate tissue and one that is very difficult to repair. The use of drugs with healing purpose must therefore be free of additional risks of tissue compromise. It is therefore of paramount importance to understand which drug class poses the least threat in this regard.

Different compounds are approved to be used for IA injections; however, it is still not clear if and how much those compounds are associated with chondrotoxicity. In this paper we aimed to review studies published regarding the possible chondrotoxic effects caused by drugs usually used for IA injections.

## 2. Materials and Methods

**Figure 1:**
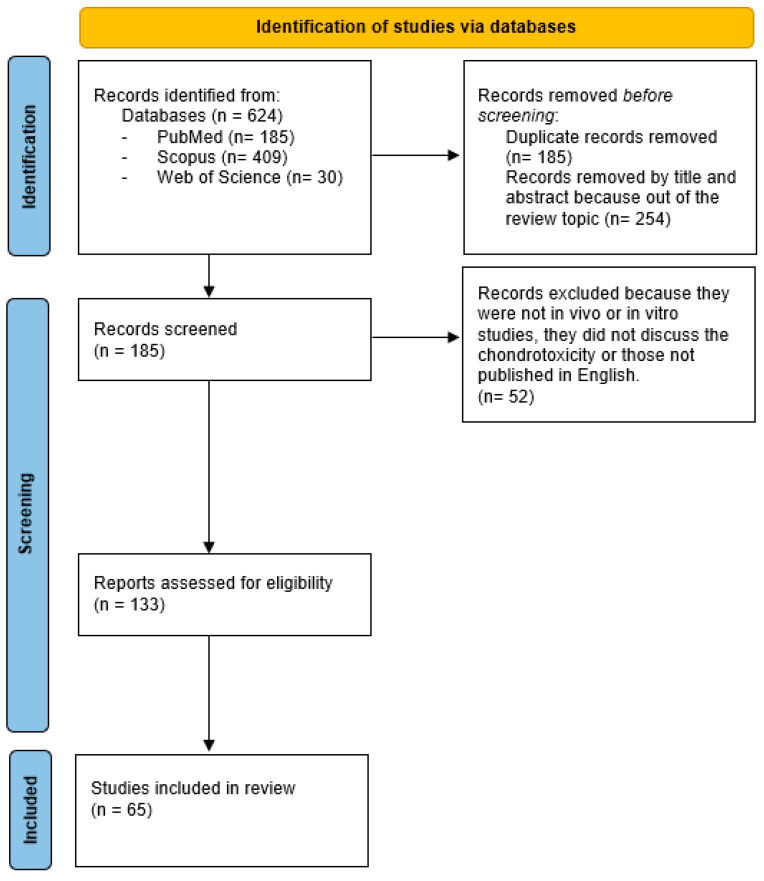
Study flow diagram.

An extensive literature search was performed using the following MeSH terms on PubMed, Scopus and Web of Science: “chondrotoxicity”, “intraarticular injection”, “corticosteroids”, “steroids”, “hyaluronate or hyaluronic acid”, “non-steroidal anti-inflammatory drug”, “anaesthetic”, “platelet rich plasma”, “collagen medical devices” and “bisphosphonates”. The search strategy was the following: “chondrotoxicity” OR (“chondrotoxicity” AND “intraarticular injection”) OR (“chondrotoxicity” AND “corticosteroids”) OR (“chondrotoxicity” AND “steroids”) OR (“chondrotoxicity” AND “hyaluronate OR hyaluronic acid”) OR (“chondrotoxicity” AND “non-steroidal anti-inflammatory drug”) OR (“chondrotoxicity” AND “anaesthetic”) OR (“chondrotoxicity” AND “platelet rich plasma”) OR (“chondrotoxicity” AND “collagen medical devices”) OR (“chondrotoxicity” AND “bisphosphonates”). The search was focused on articles published between 1975 and April 2024. Studies were included if they (1) were in vivo or in vitro studies, (2) evaluated chondrotoxicity, and (3) were published in the English language. The search was extended through the reference lists of the selected papers. The exclusion criteria encompassed (1) studies focusing on systemic delivered devices; (2) case reports, reviews and commentaries; and (3) papers not published in the English language. Articles were divided according to the delivered drug and whether they were conducted in humans or animal models. The authors looked for papers discussing the chondrotoxicity of different drugs after their IA injection. Two reviewers (C.P. and A.S.) independently selected the papers by reading titles and abstracts. A third reviewer finalized the selection in case of disagreement (A.M.). After the selection, each title/abstract/full text was independently assessed by each of the authors. The study flowchart is shown in Figure 1.

## 3. Results

In total, 65 studies were included in the review, encompassing research on 5 different compounds: corticosteroids, local anaesthetics, nonsteroidal anti-inflammatory drugs (NSAIDs), hyaluronic acids (HA) and platelet-rich plasma (PRP).

### 3.1. Corticosteroids

Papers on corticosteroids’ chondrotoxicity analysed for the purpose of this review are reported in Table 1.

A total of 33 papers regarding corticosteroids were retrieved, 14 of which utilized human subjects, 17 utilized animal subjects, and 2 investigated humans and animals in comparison. Sixteen of the papers report in vivo studies, 16 in vitro and 1 ex vivo. The knee was by far the most represented joint.

Overall, 19 (58%) studies expressed evidence about the possible chondrotoxic effects of multiple corticosteroids, raising concerns about the application of at least some of these in clinical practice. Albano et al. used multiple injections of betamethasone inside the knee of 80 living rabbits and, after 8 days of follow up, a decreased concentration of proteoglycans was found [15]. Lutfi et al. focused on betamethasone injections, also on rabbit (n = 12), for 10 weeks and, though the sample was smaller, the authors report evidence of macroscopic damage to articular cartilage [16].

Betamethasone also showed signs of chondrotoxicity in vitro. In a study on dogs’ chondrocytes, Sherman et al. used multiple drugs (1% lidocaine, 0.5% lidocaine, 0.25% bupivacaine, 0.125% bupivacaine, 0.0625% bupivacaine, betamethasone acetate, methylprednisolone acetate, and triamcinolone acetonide) finding a severe chondrotoxic effect on all of them, except bupivacaine and triamcinolone [17]. Two in vitro studies, in which multiple drugs were used (dexamethasone sodium phosphate, methylprednisolone acetate, betamethasone sodium phosphate and betamethasone acetate, or triamcinolone acetonide in combination with doses of 1% lidocaine or 0.25% bupivacaine) on human cartilage, were conducted by Braun et al. and Farkas et al. In both trials the association between corticosteroids and anaesthetic showed significant time-dependent chondrotoxicity [18,19]. Dragoo et al. also had comparable findings, with a 14-day follow-up protocol, showing significant cell death related to betamethasone use [20]. Only one study, by Davis et al., showed no harm sign with the use of betamethasone. In their in vitro study on human and animal chondrocytes, no cell death caused by the drug was reported [21].

Methylprednisolone was found to be another widely employed corticosteroid: ten studies that investigated its chondrotoxicity showed generally discouraging results. Sherman et al. [17] and Braun et al. [18] evaluated methylprednisolone among the other drugs used in their studies, confirming its detrimental effect. Sherman also conducted a second study comparing the effects of methylprednisolone and triamcinolone in association with lidocaine on 20 dogs, showing lower viable cell density 7 days after initial treatment [22]. In their 1984 study, Ishikawa et al. compared the effects of methylprednisolone with halopredone on rabbit specimens with a 13-week follow-up period and severe cartilage damages was ultimately found. This was the only study we retrieved that involved halopredone [23]. Murray et al. [24] and Pelletier et al. [25] conducted two similar studies, one regarding the effects of IA methylprednisolone on horses and the other on dogs. Both studies had an 8-week follow-up period but had the following opposite results: Murray reported alteration of the mechanical integrity of articular cartilage while Pelletier did not find any negative effect. Robion et al. also carried out a study that was analogous to Murray’s but with a longer follow up (13 weeks). This study observed the inhibition of procollagen II synthesis and increased release of degradation products of aggrecan from articular cartilage [26]. A different approach was used by Seshadri et al. who conducted an in vitro evaluation of the effects of methylprednisolone alone or in association with lidocaine [27]. The authors reported dose- and time-dependent decrease in chondrocyte viability after exposure to methylprednisolone alone and a synergistic decrease in chondrocyte survival with exposure when combined with lidocaine. An interesting paper by Chu et al. evaluated the effects of methylprednisolone (alongside naproxen and meloxicam) on an ex vivo sample of human knees. There, the authors report possible down-regulation of the plasminogen activator/plasmin system and gelatinases expression in the early osteoarthritic knee of humans, which could be related to structure-modifying activity [28]. No evidence of deleterious effects on cartilage have been reported by Gibson et al., who conducted a protocol applying methylprednisolone at the knee of 10 primates [29]. We report a final study by Baumgarten et al. which shows no evidence of chondrotoxicity in any of the 56 patients treated with methylprednisolone and anaesthetic (1% lidocaine and/or either 0.5% or 0.25% bupivacaine) [30].

Triamcinolone was also deeply analysed and we retrieved 13 papers about it, almost equally divided between those that indicated possible negative and positive effects, with a small imbalance in favour of the latter. In their multi-drugs studies, Braun et al. and Sherman et al. showed chondrotoxicity can also be caused by triamcinolone [17,18,22]. Celeste et al. focused only on triamcinolone, applying it via IA injections to horses: after a 13-week follow up, an increase in markers of cartilage matrix degradation and aggrecan turnover was found; interestingly, the authors also report altered articular cartilage and collagen metabolism in treated control joints, signs of a possible systemic effect of the IA injections [31]. Among other drugs, Dragoo et al. reported the possible chondrotoxic effect of triamcinolone [20]. Two in vitro studies were carried out by Suntiparpluacha et al. and Syed et al., applying triamcinolone on knee-derived human chondrocytes; both showed an induced chondrotoxicity and, in the first study, the authors found an increased oxidative stress and altered expressions of genes involved in cell death [32,33]. Several papers showed positive or benign effects associated with triamcinolone. Bolt et al. carried out an in vitro protocol on horses’ chondrocytes and reported that triamcinolone seems to support chondrocyte morphology in culture and protects chondrocytes from toxic effects [34]. Another study on horses was conducted by Frisbie et al., applying the drug on living animals; after a 6-week follow-up period signs of favourable effects on articular cartilage parameters were found [35]. Similarly, Pelletier et al. applied triamcinolone on 12 dogs at knee level, finding no deleterious effects on articular cartilage after 8 weeks [36]. A large study by Huppertz et al. evaluated the effect of triamcinolone on 21 humans (2 Ankle, 1 Elbow, 17 Knees). After 13 months, no evidence of toxic effects on cartilage was evident at MRI evaluation [37]. Raynauld et al. carried out an even bigger study on 68 patients who were evaluated every 3 months for 2 years after IA knee injections; at placebo comparison no difference in loss of joint space and no deleterious effects on the anatomical structure of the knee were observed [3]. Finally, Williams et al. found a marked, dose-dependent protective effect of triamcinolone applied with IA injections to the knee of 43 guinea pigs [38].

Literature is also present about the possible chondrotoxic effects of dexamethasone. We retrieved five papers, four of which reported negative outcomes. Liu et al. (humans), Song et al. (humans), Su et al. (humans and bovine) and Tu et al. (humans) used dexamethasone on knee articular cartilage samples, reporting increased apoptosis, inhibition of ECM synthesis and inhibition of positive remodelling factors (TGF-β and TIMP-3) associated with its use [39,40,41,42]. Only one paper reported a reduction of inflammatory mediator (nitric oxide) expression after application of dexamethasone on horses’ chondrocytes in vitro [43].

Finally, we retrieved three papers regarding hydrocortisone. Salter et al. have reported a deleterious effect on the articular cartilage of 55 rabbits when hydrocortisone was applied at the level of the knee [44]. On the other hand Wang et al. conducted two studies with similar characteristics (in vitro, knee-derived chondrocytes from humans) that showed enhanced ability to synthesize ECM macromolecules (aggrecan, type II collagen and fibronectin), inhibition of degenerative enzymes, increased hyaluronan levels, and inhibition of deleterious intracellular protease MMP-1 [45,46].

### 3.2. Local Anaesthetics

In our research, 18 studies regarding the chondrotoxic effects of anaesthetics were retrieved (Table 2). Papers concerned both animals and humans, in vivo and in vitro, and focused mainly on the effects of bupivacaine, lidocaine and ropivacaine.

Bupivacaine is the anaesthetic for which we found the most abundant literature. Breu et al. evaluated chondrotoxic effects of bupivacaine in comparison with ropivacaine, and mepivacaine. The authors found chondrotoxic effects in time-dependent, concentration-dependent and drug-dependent manners. In particular, chondrotoxicity increases from ropivacaine to mepivacaine to bupivacaine, indicating that chondrotoxic and analgesic potencies do not directly correlate [47]. Another in vitro study comparing the effects of bupivacaine with lidocaine and ropivacaine on bovine chondrocytes by Lo et al. reports a dose- and duration-dependent detrimental effect on chondrocyte viability [48]. An in vitro comparison on human chondrocytes between bupivacaine, ropivacaine, lidocaine and/or vitamin C was established by Tian et al., who found a chondrotoxic effect caused by all of these anaesthetics, with ropivacaine being less detrimental than bupivacaine and lidocaine; however, vitamin C improved chondrocyte viability and decreased apoptosis levels following exposure to anaesthetics [49]. Shaw et al. also compared the in vitro effects of bupivacaine, ropivacaine and liposomal Bupivacaine on bovine chondrocytes. They observed a dose-dependent chondrotoxic effect for all three drugs, with the highest chondrocyte viability associated with liposomal bupivacaine [50]. Shaw et al. also conducted a second study comparing liposomal bupivacaine and bupivacaine after IA knee injections in the knee chondrocytes of Yorkshire cross piglets and found that liposomal bupivacaine showed a good safe profile for IA injections [51]. Mwale et al. compared bupivacaine, levobupivacaine and ropivacaine in vitro on hip and elbow cells of dogs. All of the drugs caused decreased chondrocyte viability, though ropivacaine showed less chondrotoxicity [52]. We retrieved three papers by Chu et al., all of which focused on bupivacaine applied at the knee level. Two of these were in vitro studies [53,54], the first on humans and bovine chondrocytes, the second only on bovine cells. The third was an in vivo protocol on rats (n = 48) with a follow-up period of up to three months [55]. All three of Chu et al.’s papers found detrimental dose- and time-dependent effects of the drug on articular cartilage, even after only 15 to 30 min of in vitro exposure. Oyadomari et al. also carried out an in vitro study on engineered neocartilage constructs and bovine cells and have reported significant chondrotoxicity in native explants and neocartilage and a significant weakening of the mechanical properties of the neocartilage [56]. Rengert et al. evaluated the in vitro effects of bupivacaine on dog chondrocytes and have reported a concentration-dependent chondrotoxic effect [57]. Stueber et al. were the only group to compare bupivacaine and dexamethasone on human chondrocytes in vitro and found a concentration-dependent chondrotoxic effect associated with the anaesthetic, though dexamethasone failed to induce cytotoxicity [58]. Another peculiar association (bupivacaine or bupivacaine with epinephrine) was tested by Gomoll et al. on 30 live rabbits; after 1 week, significant histopathologic and metabolic changes in articular cartilage, without correlations with the drug used, were found [59]. Finally, one paper involving bupivacaine in comparison with articaine and lidocaine was carried out on the temporo-mandibular joint; 24 rabbits were subjected to IA injections and, after 4 weeks, apoptotic effects on chondrocytes and degenerative changes in the joint articular structures were found [60].

Several studies focused only on lidocaine. Karpie et al. evaluated its effects at two different concentrations (1% or 2%) in vitro on bovine knee cells and reported a dose- and time-dependent chondrotoxic effect [61]. Maeda T et al. confirmed the chondrotoxicity of lidocaine (that increased in a time- and concentration-dependent manner) in their in vitro evaluation on the knee, hip, and shoulder chondrocytes of rabbits [62]. Di Salvo et al. attempted to assess the effect of associating lidocaine plus adrenaline with IA injections in the elbows of 12 dogs. The authors report a dose- and time-dependent chondrotoxic effect of lidocaine on the viability of articular cells, reduced by the application of adrenaline [63]. Vrachnis et al. carried out the only study in which treatment did not induce any histological changes in articular cartilage. The authors applied lidocaine or ropivacaine on 32 rats and evaluated the effects after a follow-up period of up to 60 days [64].

### 3.3. Nonsteroidal Anti-Inflammatory Drugs (NSAIDs)

Only five studies were retrieved that focused specifically on the association between NSAIDs and chondrotoxicity (Table 3).

Four studies were carried out in vitro, with the only exception being the study by Sagir et al. which focused on Dexketoprofen. The authors conducted a protocol involving 35 rats with IA injections in the knee and, after a follow-up period of up to 21 days, inhibition of cell proliferation was found despite the lack of any signs of significant histopathologic effects [65].

Regarding the in vitro studies, Abrams et al. evaluated the effects on human chondrocytes related to different compound exposures and, for NSAIDs, ketorolac was associated with significantly increased cell death [66]. Alaseem et al. focused their paper on the synthesis of type X collagen (COL X), a marker of late-stage chondrocyte hypertrophy expressed in the mesenchymal stem cells (MSCs) of OA patients, their results show that naproxen seems to induce type X collagen gene (COL10A1) expression in bone-marrow-derived MSCs from healthy and OA donors, to which the authors relate as a sign of possible chondrocyte differentiation towards an undesirable degenerative phenotype [67]. In another in vitro assessment, Bèdouet et al. conclude that the limited toxicity of ibuprofen at low and high concentration in sheep joint shoulder makes this enantiomer a promising drug candidate for the loading of intra-articular DDS [68]. However, Dingle et al. report opposite results for their evaluation of 845 human cartilage samples. Here, ibuprofen, indomethacin, aspirin, naproxen and nimezulide showed substantial and significant inhibition of glycosaminoglycan synthesis while diclofenac, piroxicam, nabumetone and paracetamol had no significant effects [69].

### 3.4. Hyaluronic Acids (HAs)

For the purpose of this review, seven studies regarding the possible role of HAs on chondrotoxicity were retrieved (Table 4).

All of the studies were conducted with in vitro models and focused on assessing HA efficacy in reducing chondrotoxicity caused by other drugs. Two papers evaluated the association between HA and carprofen, both based on dog chondrocytes: in the first, Euppayo et al. report that HA alone preserved chondrocyte survival but, when in combination with carprofen, could not reduce its chondrotoxicity [70]. The second was carried out by Nganvongpanit et al., who found that HA was able to decrease chondrocyte apoptosis [71]. Euppayo et al. also evaluated HA in association with a corticosteroid, triamcinolone. The authors applied the drugs in vitro on dog knee and elbow-derived chondrocytes and have reported that the combination increased the percentage of cell viability in normal chondrocytes [72].

Four studies analysed the association of HA with anaesthetics. Its association with lidocaine was assessed by Lee et al. The authors report that HA seems to suppress lidocaine-induced apoptosis of human chondrocytes in vitro [73]. Liu et al. evaluated its association with bupivacaine. In their paper, bovine knee-derived chondrocytes were used, with the results showing a reduction of cell death rates [74]. Bovine chondrocytes were also used by Onur et al. to test HA effects in association with lidocaine and bupivacaine; HA was able to reduce cytotoxicity caused by the second but not by the first [75]. The last paper we retrieved, by Moser et al., was conducted to test the effects of HA in association with anaesthetics (lidocaine, bupivacaine, ropivacaine), and glucocorticoids. Human knee derived chondrocytes were used and the authors report that HA enhanced attachment and branched appearance of the chondrocytes and improved the metabolic activity [76].

### 3.5. Platelet-Rich Plasma (PRP)

Few studies have assessed the efficacy and safety of infiltrative treatment with PRP; for this paper we were able to find only two (Table 5).

In fact, in 2015 Al-Ajilouni et al. observed that IA PRP injections significantly improved functional outcome scores in 48 patients with knee osteoarthritis after a follow-up period of 52 weeks. There was no specific association and a specific paragraph on chondrotoxicity was not present, although it is likely that the improvement in functional outcomes could be directly related to a corresponding improvement in articular cartilage qualities [77]. In 2013 Beitzel et al. focused their work specifically on PRP-related chondrotoxicity; their study showed that tendon and cartilage cells increased cell viability after an exposure to allogeneic PRP. Proliferation also increased [78].

### 3.6. Collagen Medical Devices, Biphosphonates

No papers were retrieved regarding collagen medical devices (CMDs) or for bisphosphonates.

## 4. Discussion

A wide variety of protocols were found for all samples and settings, with heterogeneous results. This lack of homogeneity makes it complicated to perform comparative analyses either between studies involving the same molecule or, even more, between different compounds. Thus, all the possible conclusions we could draw are weaker and the answer to the question posed at the beginning still seems unanswered.

Corticosteroids are among the most used drugs for IA injection in consideration of their well-known anti-inflammatory properties (i.e., capacity to reduce the inflammatory cells inside joints, to prevent phagocytosis, lysosomal enzyme release, and to inhibit pro-inflammatory cytokines). Thus, IA corticosteroids are able to reduce pain and swelling and increase joint range of motion (ROM) and functionality by decreasing inflammation [79,80].

These pharmacological classes can improve functional outcomes for degenerative diseases or after an injury and can reduce the time to return to a good quality of life (QoL) for people affected by different painful conditions [81,82,83,84]. However, an abundance of data are available in the literature about their possible toxic effects on articular cartilage. Both direct and indirect evidence for reduced articular cartilage matrix synthesis and/or proteoglycan degradation have been provided both by in vitro and in vivo studies [85]. Although there is general agreement on their dose- or time-dependent toxicity, they are still widely used for IA injection. If, on one hand, ACR guidelines [86] “…recommends intraarticular corticosteroid injections for the knee and hip…”, on the other hand both OARSI and AAOS underline how intra-articular steroid therapy is advised with a low level of evidence [4,87].

Studies have been conducted on both animal and humans, both in vivo and in vitro. In 2015 Wernecke et al. published a systematic review on the effect of intra-articular corticosteroids on articular cartilage. Forty scientific papers were selected out of 1929 publications on the topic and the collected data were divided on the basis of the investigated steroid formulation. The authors concluded that the time- and dose-dependent deleterious effects on articular cartilage are widely supported by the basic scientific literature. [88]. Our results seem to support these findings; most of the papers we assessed reported risks of chondrotoxicity, with triamcinolone appearing to be the safest intra-articular corticosteroids, though only because the other analysed seemed to be even worse.

Almost all of the papers about local anaesthetics showed signs of chondrotoxicity, and investigated them via conventional histologic analysis, cell quantification (confocal, immunofluorescence), nuclear morphology changes, and metabolic sulphate uptake assessment. These studies were mainly focused on the effects of bupivacaine, lidocaine and ropivacaine. Local anaesthetics are widely used to achieve analgesia in painful joints. Anaesthetics are usually injected inside the knee, shoulder or other joints, often combined with other agents, such as corticosteroids, after injury or for the treatment of degenerative disease. Local anaesthetics express their effects by inhibiting voltage-gated sodium channels on nerve cell membranes, thereby preventing development of an action potential and blocking nerve transmission [89]. Usually, a single dose of an anaesthetic is enough to achieve pain control after IA injections, though this also depends on the properties of the single molecule (e.g., bupivacaine, ropivacaine, lidocaine) [90,91]. Achieving a rapid reduction of pain is crucial for the success of an individual rehabilitation project (IRP). Local anaesthetics seem to have an overall detrimental effect on articular cartilage, with a dose-and time-dependent modality. Ropivacaine appears to be the safer of these, alongside liposomal bupivacaine. The association with vitamin C seems to reduce the negative effects but further research is needed.

NSAIDs are widely used in arthritis and osteoarthritis, mostly to reduce pain caused by the disease and relative functional burdens [92,93]. NSAIDs inhibit cyclooxygenase (COX-1 and COX-2), thus preventing the synthesis of inflammation mediators (e.g., prostaglandin, prostacyclin and thromboxane). Some of the most used NSAIDs (i.e., naproxen, diclofenac, aspirin and ibuprofen) non-selectively inhibit both COX-1 and COX-2, though selective COX-2 inhibitors are also available, such as celecoxib and meloxicam [94]. This difference is not a mere pharmacological one, it has several clinical implications. COX-1 is expressed in most tissues and has a crucial role in the protection of the gastric mucosa, in the regulation of the renal blood flow and of the vascular homeostasis. COX-2 is less constitutively expressed but higher concentrations can be found near inflamed tissues [95]. Numerous ways of administration are available for NSAIDs: oral, intradermal, intravenous and intramuscular; IA injections with NSAIDs are not widely used though this method of delivery might reduce systemic side effects while at the same time increasing local efficacy [96]. NSAIDs have also shown a rather low safety profile: 4 of the 5 studies analysed indicated various types of damage to articular cartilage. The one exception indicated encouraging results for only some molecules (aceclofenac, tenidap and tolmetin) and still reported problems with others (ibuprofen, indomethacin, aspirin, naproxen and nimezulide). Data about the NSAIDs use for IA injections and about their chondrotoxicity are, however, too scarce to be relied upon to finalize any conclusion. Broader evaluation must thus be carried out.

A different point can be raised, on the other hand, for HA. HA is one of the most used compounds for IA injections, especially for degenerative diseases such as osteoarthritis, which causes functional disability and reduction of QoL. HA is physiologically present inside joints, both at the cartilage and the synovial fluid level [97]. HA has the important role of lubricant and homeostasis regulator inside joints; with disease progression its properties change and mechanical abnormalities in the synovial fluid arise [98]. Viscosupplementation with HA could thus be helpful to restore a more physiological microenvironment inside affected joints. Two peculiarities were noticed with regard to the studies we retrieved about HA: all were conducted in vitro and all focused on assessing their efficacy in reducing other compounds’ chondrotoxicity. Overall, HA seems to have good efficacy in reducing the damaging effects on articular cartilage caused by other molecules. No study was retrieved about the possible effects of HA alone; chances are that its properties are not related with chondrotoxicity, but research would be needed to confirm this hypothesis. 

Finally, of the two studies we assessed regarding PRP, only one was actually focused on chondrotoxicity. PRP is a treatment that uses platelet-enriched blood plasma to promote tissue regeneration. Platelets contain growth factors, which are essential for wound healing and tissue repair. These agents, alongside many others (i.e., transforming growth factor b, fibroblast growth factor, vascular endothelial growth factor, and connective tissue growth factor) account for the therapeutic effects of PRP [99]. PRP is obtained by centrifuging the patient’s own blood, and then injected into the area to be treated to stimulate healing and regeneration. PRP is emerging as a non-surgical option for degenerative diseases of the musculoskeletal system, as it carries the possibility of reversing cartilage damage, joint space thinning and associated pain [100,101]. The risks of adverse reactions related to the treatment are reduced by it being autologous [102]. However, the literature evidently lacks papers on this topic; any kind of consideration made at this time seems to be inadequate and more research is needed to clarify PRP potential, both beneficial and harmful.

We found no information regarding the chondrotoxic effects of CMDs and bisphosphonates. This undoubtedly represents a gap in the literature and makes it impossible to draw any conclusions about these compounds. This finding could be a stimulus for future research in this regard.

## 5. Conclusions

In conclusion, current research regarding the chondrotoxicity of the most commonly used drugs for IA injection lacks the standardization and numerosity to allow definitive assumptions. Corticosteroids (with the possible exception of triamcinolone), local anaesthetics (with the possible exception of ropivacaine and liposomal bupivacaine), and NSAIDs showed too poor a safety profile to be used lightly in clinical practice. HA appear to be safe and also able to reduce the risks associated with other compounds, thus favouring therapeutic combinations. Moreover, too few data exist with regard to PRP, the potential of which has yet to be evaluated. Finally, it appears that results are heavily dependent on dosage and number of injections used, both on humans and animals. In our opinion, a careful clinical application that falls within the framework of a set of guidelines may prevent side effects and adverse events.

## Figures and Tables

**Table 1:** Corticosteroids.

Authors	Title	Drug(s)	Marker	Joint	Animal	Numerosity	Follow Up	Results
Raynauld JP et al., 2003 [3]	Safety and efficacy of long-term intra-articular steroid injections in osteoarthritis of the knee: a randomized, double-blind, placebo-controlled trial	Triamcinolone acetonide 40 mg or placebo injections	In vivo	Knee	Humans	68	Every 3 months for 2 years	No difference in loss of joint space was observed at the 1- or 2-year follow up evaluations. No deleterious effects of the long-term administration of IA steroids on the anatomical structure of the knee
Albano MB et al., 2009 [15]	Computerized photocolorimetric analysis of the effects of intra-articular betamethasone on the proteoglycan concentration of leporine knee cartilage matrix: influence of the number of intra-articular injections	Repeated injections of betamethasone	In vivo	Knee	Animals (rabbits)	80	8 days	Decrease in the concentration of articular cartilage proteoglycans
Lutfi AM et al., 1978 [16]	Effects of intra-articularly administered corticosteroids and salicylates on the surface structure of articular cartilage	Repeated intra-articular corticosteroid and salicylate injections	In vivo	Knee	Animals (rabbit)	12	10 weeks	Corticosteroid-treated articular cartilage exhibits progressive lesions, including fissuring and fraying
Sherman L et al., 2015 [17]	In vitro toxicity of local anaesthetics and corticosteroids on chondrocyte and synoviocyte viability and metabolism	1% lidocaine, 0.5% lidocaine, 0.25% bupivacaine, 0.125% bupivacaine, 0.0625% bupivacaine, betamethasone acetate, methylprednisolone acetate, triamcinolone acetonide	In vitro	/	Animals (dogs)	/	7 Days	1% and 0.5% lidocaine, 0.25% and 0.125% bupivacaine, betamethasone acetate, and methylprednisolone acetate were severely chondrotoxic. No chondrotoxicity in samples exposed to 0.0625% bupivacaine and triamcinolone remained
Braun J et al., 2012 [18]	The effect of local anaesthetic and corticosteroid combinations on chondrocyte viability	Single injection doses of 1% lidocaine or 0.25% bupivacaine in combination with single injection doses of dexamethasone sodium phosphate, methylprednisolone acetate, betamethasone sodium phosphate and betamethasone acetate, or triamcinolone acetonide	In vitro	/	Humans	/	14 Days	significant chondrotoxicity when compared with the local anaesthetics alone
Farkas B et al., 2010 [19]	Increased chondrocyte death after steroid and local anaesthetic combination	Different solutions alone (betamethasone, prednisolone, lidocaine, bupivacaine, and ropivacaine) or in combinations (betamethasone with lidocaine, betamethasone with bupivacaine, betamethasone with ropivacaine, prednisolone with lidocaine)	In vitro, ex vivo	Human chondrocytes; Osteochondral specimens	Humans	/	/	Time-dependent decrease in chondrocyte viability after concurrent steroid and local anaesthetic exposure
Dragoo JL et al., 2012 [20]	The chondrotoxicity of single-dose corticosteroids.	Single-injection doses of dexamethasone sodium phosphate, methylprednisolone acetate, betamethasone sodium phosphate, betamethasone acetate, and triamcinolone acetonide	In vitro	Human chondrocytes	Humans	/	14 days	triamcinolone acetonide may be chondrotoxic. Betamethasone sodium phosphate and betamethasone acetate, significant cell death
Davis D et al., 2010 [21]	In vitro cytotoxic effects of benzalkonium chloride in corticosteroid injection suspension	Betamethasone sodium phosphate, betamethasone acetate, and benzalkonium chloride	In vitro	Human and bovine articular chondrocytes, bovine synovial cells, mouse C3H10T1/2 cells, and human osteosarcoma MG-63 cells	Humans and animals	One human donor, multiple animals	/	No cell death caused by betamethasone Cell death caused by benzalkonium chloride.
Sherman L et al., 2015 [22]	In vivo toxicity of local anaesthetics and corticosteroids on chondrocyte and synoviocyte viability and metabolism	methylprednisolone/1.0% lidocaine, triamcinolone/1.0% lidocaine, and triamcinolone/0.0625% bupivacaine	In vivo	Shoulder	Animals (dogs)	20	7 Days	After 7 days of culture, cartilage viable cell density was significantly lower
Ishikawa K et al., 1984 [23]	Effects of intra-articular injection of halopredone diacetate on the articular cartilage of rabbit knees: a comparison with methylprednisolone acetate	Halopredone diacetate and methylprednisolone acetate	In vivo	Knee	Animals (rabbit)	/	13 weeks	Severe cartilage lesions
Murray RC et al., 1998 [24]	The effects of intra-articular methylprednisolone and exercise on the mechanical properties of articular cartilage in the horse	Methylprednisolone acetate	In vivo	Middle carpal joint	Animals (horses)	8	8 weeks	Methylprednisolone acetate alters the mechanical integrity of articular cartilage
Pelletier JP et al., 1994 [25]	Intra-articular injections with methylprednisolone acetate reduce osteoarthritic lesions in parallel with chondrocyte stromelysin synthesis in experimental osteoarthritis	Intra-articular injections of methylprednisolone acetate	In vivo	Knee	Animals (dogs)	15	8 weeks	No deleterious effects on normal articular cartilage
Robion FC et al., 2000 [26]	Use of synovial fluid markers of cartilage synthesis and turnover to study effects of repeated intra-articular administration of methylprednisolone acetate on articular cartilage in vivo	Intra-articular injections of methylprednisolone acetate	In vivo	Radiocarpal	Animals (horses)	10	13 weeks	Potentially harmful inhibition of procollagen II synthesis and increased release of degradation products of aggrecan from articular cartilage
Seshadri V et al., 2009 [27]	Lidocaine potentiates the chondrotoxicity of methylprednisolone	Chondrocytes exposed in vitro to clinical doses of methylprednisolone and methylprednisolone with lidocaine	In vitro	/	Animals (bovine)	/	/	Dose- and time-dependent decrease in chondrocyte viability after exposure to methylprednisolone treatment. Synergistic decrease in chondrocyte survival with exposure to methylprednisolone combined with lidocaine
Chu SC et al., 2008 [28]	Naproxen, meloxicam and methylprednisolone inhibit urokinase plasminogen activator and inhibitor and gelatinases expression during the early stage of osteoarthritis	Naproxen, meloxicam and methylprednisolone	Ex vivo	Knee	Humans	5	4 days	Naproxen, meloxicam and methylprednisolone can downregulate the plasminogen activator/plasmin system and gelatinases expression in the early osteoarthritic knee of humans: potential structure-modifying activity
Gibson T et al., 1977 [29]	Effect of intra-articular corticosteroid injections on primate cartilage	one, two, or six injections of 20 mg methyl prednisolone	In vivo	Knee	Animals (primate)	10	12 weeks	No evidence of a deleterious effect on primate cartilage by corticosteroids
Baumgarten et al., 2015 [30]	Does chondrolysis occur after corticosteroid analgesic injections? An analysis of patients treated for adhesive capsulitis of the shoulder	Methylprednisolone acetate) and anaesthetic (1% lidocaine and/or either 0.5% or 0.25% bupivacaine)	In vivo	Shoulder	Humans	56	54 months	No evidence of chondrolysis in any patient
Celeste C et al., 2004 [31]	Repeated intra-articular injections of triamcinolone acetonide alter cartilage matrix metabolism measured by biomarkers in synovial fluid	3 triamcinolone injection (12 mg)	In vivo	Radiocarpal	Animals (horses)	10	13 weeks	The trial showed an increase in markers of cartilage matrix degradation and aggrecan turnover. Altered articular cartilage and collagen metabolism in treated control joints
Suntiparpluacha M et al., 2016 [32]	Triamcinolone acetonide reduces viability, induces oxidative stress, and alters gene expressions of human chondrocytes	Triamcinolone acetonide	In vitro	Knee	Humans	10 donors	14 days	Induced chondrotoxicity by increasing oxidative stress and altering expressions of genes involved in cell death
Syed HM et al., 2011 [33]	Bupivacaine and triamcinolone may be toxic to human chondrocytes: a pilot study	Bupivacaine and triamcinolone acetonide	In vitro	Knee	Humans	3	/	Induced chondrotoxicity
Bolt DM et al., 2008 [34]	Effects of triamcinolone acetonide, sodium hyaluronate, amikacin sulphate, and mepivacaine hydrochloride, alone and in combination, on morphology and matrix composition of lipopolysaccharide-challenged and unchallenged equine articular cartilage explants	Articular cartilage explants treated with triamcinolone	In vitro	96 Articular cartilage explants from four femoropatellar joints	Animals (horses)	2	/	Triamcinolone supports chondrocyte morphology in culture and protects chondrocytes from toxic effects
Frisbie DD et al., 1997 [35]	Effects of triamcinolone acetonide on an in vivo equine osteochondral fragment exercise model	Triamcinolone acetonide	In vivo	Equine carpal osteochondral fragment	Animals (horses)	18	6 weeks	Favourable effects of triamcinolone acetonide on articular cartilage parameters
Pelletier JP et al., 1989 [36]	Protective effects of corticosteroids on cartilage lesions and osteophyte formation in the Pond–Nuki dog model of osteoarthritis	Intra-articular injections of triamcinolone hexacetonide	In vivo	Knee	Animals (dogs)	12	8 weeks	No deleterious effects on normal articular cartilage
Huppertz HI et al., 1995 [37]	Intra-articular corticosteroids for chronic arthritis in children: efficacy and effects on cartilage and growth	Triamcinolone hexacetonide in children with chronic arthritis	In vivo	Ankle (2), elbow (1), knee (17)	Humans	21	13 months	No evidence of toxic effects on cartilage at MRI
Williams JM et al., 1985 [38]	Triamcinolone hexacetonide protects against fibrillation and osteophyte formation following chemically induced articular cartilage damage	Triamcinolone hexacetonide	In vivo	Knee	Animals (guinea pigs)	43	/	Marked, dose-dependent protective effect
Liu N et al., 2014 [39]	Autophagy in human articular chondrocytes is cytoprotective following glucocorticoid stimulation	Dexamethasone	In vitro	Knee	Humans	10	72 h	Prolonged dexamethasone treatment increases apoptosis
Song YW et al., 2012 [40]	Gluococorticoid could influence extracellular matrix synthesis through Sox9 via p38 MAPK pathway	Dexamethasone	In vitro	Knee	Humans	Knee joint sample from 9 patients	Up to 1 week	Inhibition of extracellular matrix synthesis
Su S et al., 1996 [41]	Regulation of tissue inhibitor of metalloproteinases-3 gene expression by transforming growth factor-b and dexamethasone in bovine and human articular chondrocytes	Dexamethasone	In vitro	Knee	Humans and animals (bovine)	/	/	Inhibition of positive remodelling factors (TGF-β and TIMP-3)
Tu Y et al., 2013 [42]	Lactoferrin inhibits dexamethasone-induced chondrocyte impairment from osteoarthritic cartilage through upregulation of extracellular signal-regulated kinase 1/2 and suppression of FASL, FAS, and Caspase 3	Dexamethasone	In vitro	Knee	Humans	8	/	Detrimental effect on chondrocytes proliferation, viability, and apoptosis
Tung JT et al., 2002 [43]	Inducible nitric oxide expression in equine articular chondrocytes: effects of anti-inflammatory compounds	Dexamethasone	In vitro	Metacarpo-phalangeal Joint	Animals (horses)	/	/	Reduced inflammatory mediators (NO) expression
Salter RB et al., 1967 [44]	Hydrocortisone arthropathy—an experimental investigation	Repeated intra-articular injection of hydrocortisone	In vivo	Knee	Animals (rabbit)	55	11 weeks	Deleterious effect on the articular cartilage
Wang J et al., 2004 [45]	Physiological levels of hydrocortisone maintain an optimal chondrocyte extracellular matrix metabolism	Hydrocortisone	In vitro	Knee	Humans	5 donors	/	Enhanced ability to synthesise ECM macromolecules (aggrecan, type II collagen and fibronectin) and inhibition of degenerative enzymes.
Wang L et al., 2002 [46]	Influence of polysulphated polysaccharides and hydrocortisone on the extracellular matrix metabolism of human articular chondrocytes in vitro.	Hydrocortisone	In vitro	Knee	Humans	7 donors	/	Accumulation of ECM macromolecules (aggrecan), increased hyaluronan levels, and inhibition of deleterious intracellular protease MMP-1

**Table 2:** Local anaesthetics.

Authors	Title	Drug(s)	Marker	Joint	Animal	Numerosity	Follow Up	Results
Breu A et al., 2013 [47]	The cytotoxicity of bupivacaine, ropivacaine, and mepivacaine on human chondrocytes and cartilage	Bupivacaine (0.5%), ropivacaine (0.75%) and mepivacaine (2%)	In vitro	Femoral	Humans	4 donors	Up to 7 days	Time-dependent and concentration-dependent chondrotoxic effects
Lo IK et al., 2009 [48]	Local anaesthetics induce chondrocyte death in bovine articular cartilage disks in a dose- and duration-dependent manner	0.25% bupivacaine, 1% lidocaine, and 0.5% ropivacaine	In vitro	Radial-carpal joint	Animal (bovine)	/	12 h	Dose- and duration-dependent detrimental effect on chondrocyte viability
Tian J et al., 2016 [49]	Comparative effects of vitamin C on the effects of local anaesthetics ropivacaine, bupivacaine, and lidocaine on human chondrocytes.	Bupivacaine (0.5%) or ropivacaine (0.75%) or lidocaine (1.0%) and/or vitamin C at doses of 125, 250 and 500 μM	In vitro	Normal human chondrocytes cell line	Humans	/	/	Chondrotoxic effect caused by all anaesthetics (ropivacaine < bupivacaine < lidocaine). Vitamin C improved chondrocyte viability and decreased apoptosis levels following exposure to anaesthetics
Shaw KA et al., 2017 [50]	Chondrotoxicity of liposomal bupivacaine in articular chondrocytes: preliminary findings	Bupivacaine or ropivacaine (0.5%, 0.25%, and 0.13%) and liposomal bupivacaine (1.3%)	In vitro	Stifle joint	Animals (bovine)	/	/	Dose-dependent chondrotoxic effect. Highest chondrocyte viability associated with liposomal bupivacaine
Shaw KA et al., 2018 [51]	Improved chondrotoxic profile of liposomal bupivacaine compared with standard bupivacaine after intra-articular infiltration in a porcine model	1.3% liposomal bupivacaine or 0.5% bupivacaine	In vivo	Knee	Animal (Yorkshire cross piglets)	8	1 week	Decreased chondrocyte viability on a cellular level but no histological changes. Liposomal bupivacaine showed a good safe profile for intra-articular injection
Mwale C et al., 2023 [52]	In vitro chondrotoxicity of bupivacaine, levobupivacaine and ropivacaine and their effects on caspase activity in cultured canine articular chondrocytes	Bupivacaine, levobupivacaine and ropivacaine (all 0.062%)	In vitro	Hip, elbow	Animal (dogs)	2 donors	/	Decreased chondrocyte viability by all drugs used, ropivacaine showed less chondrotoxicity
Chu CR et al., 2008 [53]	The in vitro effects of bupivacaine on articular chondrocytes	Bupivacaine	In vitro	Knee	Humans and Animals (bovine)	3 human donors and 5 bovine	24 h	Dose- and time-dependent chondrotoxic effect on both humans and bovine cartilage
Chu CR et al., 2006 [54]	In vitro exposure to 0.5% bupivacaine is cytotoxic to bovine articular chondrocytes	Bupivacaine	In vitro	Knee	Animal (bovine)	12	Up to 24 h	Chondrotoxic effect on chondrocytes and articular cartilage in vitro after only 15 to 30 min exposure
Chu CR et al., 2010 [55]	In vivo effects of single intra-articular injection of 0.5% bupivacaine on articular cartilage	Bupivacaine	In vivo	Knee	Animal (rats)	48	Up to 6 months	Reduced chondrocyte density without cartilage tissue loss at the six months follow up time point
Oyadomari S et al., 2021 [56]	In vitro effects of bupivacaine on the viability and mechanics of native and engineered cartilage grafts	Bupivacaine	In vitro	Knee and engineered neocartilage constructs	Animal (bovine)	/	Up to 6 days	Significant chondrotoxicity in native explants and neocartilage and significant weakening of mechanical properties of neocartilage
Rengert R et al., 2021 [57]	Effect of bupivacaine concentration and formulation on canine chondrocyte viability in vitro	Bupivacaine	In vitro	Frozen canine articular chondrocytes	Animal (dogs)	/	/	Concentration-dependent chondrotoxic effect
Stueber T et al., 2014 [58]	Differential cytotoxic properties of drugs used for intra-articular injection on human chondrocytes. An experimental in vitro study	Bupivacaine and dexamethasone	In vitro	Cartilage cell line T/C 28-a2	Humans	/	/	Bupivacaine: concentration-dependent chondrotoxic effect Dexamethasone failed to induce cytotoxicity
Gomoll AH et al., 2006 [59]	Chondrolysis after continuous intra-articular bupivacaine infusion: an experimental model investigating chondrotoxicity in the rabbit shoulder	Bupivacaine or bupivacaine with epinephrine	In vivo	Gleno-humeral	Animal (rabbits)	30	1 week	Significant histopathologic and metabolic changes in articular cartilage without correlations with the drug used
Asan CY et al., 2022 [60]	Chondrotoxic effects of intra-articular injection of local anaesthetics in the rabbit temporomandibular joint	Articaine, lidocaine, and bupivacaine	In vivo	Temporo-mandibular joint	Animal (rabbits)	24	4 weeks	Apoptotic effects on chondrocytes, degenerative changes in the temporo-mandibular joint articular structures. Less harmful effects by Articaine
Karpie JC et al., 2007 [61]	Lidocaine exhibits dose- and time-dependent cytotoxic effects on bovine articular chondrocytes in vitro	1% or 2% lidocaine	In vitro	Knee	Animal (bovine)	/	1 week	Dose- and time-dependent chondrotoxic effect
Maeda T et al., 2015 [62]	Lidocaine Induces ROCK-dependent membrane blebbing and subsequent cell death in rabbit articular chondrocytes	Lidocaine (3–30 mM)	In vitro	Knee, hip, and shoulder	Animal (rabbit)	/	/	Time- and concentration-dependent chondrotoxic effect
Di Salvo et al., 2015 [63]	Intra-articular administration of lidocaine plus adrenaline in dogs: Pharmacokinetic profile and evaluation of toxicity in vivo and in vitro	Lidocaine (L) 1.98% plus adrenaline 1:100.000	In vivo	Elbow	Animal (dogs)	12	/	Dose- and time-dependent chondrotoxic effect of lidocaine on the viability of articular cells, reduced by adrenaline
Vrachnis et al., 2024 [64]	The in vivo chondrotoxicity of single intra-articular injection of local anaesthetic in rat cartilage	Lidocaine (2%) or ropivacaine (0.75%)	In vivo	Knee	Animal (rats)	32	Up to 60 Days	Treatment did not induce any histological changes in the rat cartilage

**Table 3:** NSAIDs.

Authors	Title	Drug(s)	Marker	Joint	Animal	Numerosity	Follow Up	Results
Sagir O et al., 2013 [65]	Evaluation of the effects of dexketoprofen trometamol on knee joınt: an in vivo and in vitro study	Dexketoprofen trometamol	In vivo	Knee	Animals (rats)	35	Up to 21 days	No significant histopathologic effects. Cell proliferation was inhibited.
Abrams GD et al., 2017 [66]	In vitro chondrotoxicity of nonsteroidal anti-inflammatory drugs and opioid medications	Morphine sulphate (0.01%, 0.02%, and 0.04%). Ketorolac tromethamine (0.3% and 0.6%). Meperidine hydrochloride (0.5%, 1.0%, and 1.5%). Fentanyl citrate (and 0.0005% and 0.001%).	In vitro	Knee	Humans	16	Up to 2 weeks	Ketorolac caused significantly increased cell death versus the saline control
Alaseem AM et al., 2015 [67]	Naproxen induces type X collagen expression in human bone-marrow-derived mesenchymal stem cells through the upregulation of 5-lipoxygenase	Naproxen sodium	In vitro	Normal human MSCs	Humans	7	72 h	Naproxen induces chondrocyte differentiation towards an undesirable degenerative phenotype in normal and OA hMSCs
Bèdouet L et al., 2011 [68]	In vitro evaluation of (S)-ibuprofen toxicity on joint cells and explants of cartilage and synovial membrane	Ibuprofen	In vitro	Shoulder	Animals (sheep)	3	Up to 13 days	Ibuprofen did not reduce cell viability and protein content on chondrocyte monolayers. Ibuprofen reduced synoviocyte viability. Overall, ibuprofen showed limited toxicity.
Dingle JT et al., 1999 [69]	The effects of NSAID on the matrix of human articular cartilages	Aceclofenac, tenidap, tolmetin, ibuprofen, indomethacin, aspirin, naproxen, nimezulide, diclofenac, piroxicam, nabumetone and paracetamol	In vitro	Femoral head	Humans (in vitro)	845	/	Aceclofenac, tenidap and tolmetin: significant stimulation of glycosaminoglycan synthesis. Ibuprofen, indomethacin, aspirin, Naproxen and nimezulide: substantial and significant inhibition of glycosaminoglycan synthesis. Diclofenac, piroxicam, nabumetone and paracetamol: no significant effects.

**Table 4:** Hyaluronic acids.

Authors	Title	Drug(s)	Marker	Joint	Animal	Numerosity	Follow Up	Results
Euppayo T et al., 2015 [70]	Effects of low molecular weight hyaluronan combined with carprofen on canine osteoarthritis articular chondrocytes and cartilage explants in vitro	Hyaluronic acid + carprofen	In vitro	/	Animals (dogs)	/	7 days	Hyaluronic acid alone preserved chondrocyte survival. In combination with carprofen, hyaluronic acid could not reduce the chondrotoxicity of carprofen.
Nganvongpanit K et al., 2020 [71]	Post-treatment of hyaluronan to decrease the apoptotic effects of carprofen in canine articular chondrocyte culture	Hyaluronic acid + carprofen	In vitro	/	Animals (dogs)	3	/	Decreased chondrocytes’ apoptosis if hyaluronic acid is administered 24 h after carprofen
Euppayo T et al., 2016 [72]	In vitro effects of triamcinolone acetonide and in combination with hyaluronan on canine normal and spontaneous osteoarthritis articular cartilage	Hyaluronic acid + triamcinolone acetonide	In vitro	Knee and elbow	Animals (dogs)	/	/	The combination of HA and TA increased the percentage of cell viability in normal chondrocytes
Lee YJ et al., 2020 [73]	Hyaluronan suppresses lidocaine-induced apoptosis of human chondrocytes in vitro by inhibiting the p53-dependent mitochondrial apoptotic pathway	Hyaluronic acid + lidocaine	In vitro	/	Humans and animals (mouse)	3	72 h	Hyaluronan suppresses lidocaine-induced apoptosis of human chondrocytes in vitro
Liu S et al., 2012 [74]	Hyaluronan protects bovine articular chondrocytes against cell death induced by bupivacaine at supraphysiologic temperatures	Hyaluronic acid + bupivacaine	In vitro	Knee	Animals (bovine)	6	Up to 24 h	Reduced chondrocytes’ cell death rates
Onur TS et al., 2013 [75]	Co-administration of hyaluronic acid with local anaesthetics shows lower cytotoxicity than local anaesthetic treatment alone in bovine articular chondrocytes	Hyaluronic acid + lidocaine, bupivacaine	In vitro	Knee	Animals (bovine)	/	/	Reduced cytotoxicity caused by anaesthetic with bupivacaine but not lidocaine
Moser LB et al., 2015 [76]	Hyaluronic acid as a carrier supports the effects of glucocorticoids and diminishes the cytotoxic effects of local anaesthetics in human articular chondrocytes in vitro	Hyaluronic acid + lidocaine, bupivacaine, ropivacaine, and glucocorticoids	In vitro	Knee	Humans	5 donors	/	Hyaluronic acid enhanced attachment and branched appearance of the chondrocytes and improved metabolic activity

**Table 5:** Platelet-Rich Plasma (PRP).

Authors	Title	Drug(s)	Marker	Joint	Animal	Numerosity	Follow Up	Results
Al-Ajlouni J et al., 2015 [77]	Safety and efficacy of autologous intra-articular platelet lysates in early and intermediate knee osteoarthrosis in humans: a prospective open-label study	PRP	In vivo	Knee	Humans	48 patients	Up to 52 weeks	Improvement of functional outcomes scores. Platelet lysate seems to have a positive influence on early and intermediate KAO. The improvement in KOOS started as early as 12 weeks after the first injection. More solid improvement in all the points scored by KOOS showed statistically significant improvement at week 32 and more so at week 52. As for the safety, we observed three intra-articular bleeding episodes: one after the second injection and two after the third injection. One of the episodes was mild and settled with simple analgesia and two required an overnight hospitalization for observation. It is not known how much of this is due to technical cause or due to the PL itself. No additional adverse reactions, such as acute pain, swelling or major complications, such as infection, were noted.
Beitzel K et al., 2013 [78]	The Effect of ketorolac tromethamine, methylprednisolone, and platelet-rich plasma on human chondrocyte and tenocyte viability	PRP, Ketorolac tromethamine, and methylprednisolone	In vitro	/	Humans	8 PRP donors. chondrocyte cell line	120 h	Increased cell viability after an exposure to allogeneic PRP and/or ketorolac tromethamine (the cell number was highest for the combined treatment). No significant changes evoked by methylprednisolone; positive effects if administered in association with PRP

## Data Availability

The data presented in this study are available on request from the corresponding author. The data are not publicly available due to privacy.
